# Follicular growth after xenotransplantation of cryopreserved/thawed human ovarian tissue in SCID mice: dynamics and molecular aspects

**DOI:** 10.1007/s10815-016-0769-2

**Published:** 2016-07-27

**Authors:** Sarrah Ayuandari, Katharina Winkler-Crepaz, Monika Paulitsch, Cora Wagner, Claudia Zavadil, Claudia Manzl, Stephanie C. Ziehr, Ludwig Wildt, Susanne Hofer-Tollinger

**Affiliations:** 1 Department of Gynecological Endocrinology and Reproductive Medicine, Medical University of Innsbruck, Anichstr. 35, 6020 Innsbruck, Austria; 2 Department of Obstetrics and Gynecology, Faculty of Medicine, Gadjah Mada University, Jl. Farmako, 55281 Yogyakarta, Indonesia; 3 Institute of Zoology, University of Innsbruck, Technikerstr. 25, 6020 Innsbruck, Austria; 4 Department of Pathology, Medical University of Innsbruck, Müllerstr. 44, 6020 Innsbruck, Austria; 5 A.R.T. Bogenhausen, Prinzregentenstr. 69, 81675 Munich, Germany

**Keywords:** Fertility preservation, Human ovarian tissue xenotransplantation, Human ovarian tissue cryopreservation, Follicular recruitment, PTEN

## Abstract

**Purpose:**

To study the influence of xenotransplantation on follicular recruitment and growth in cryopreserved/thawed human ovarian tissue.

**Method:**

Two 3-mm pieces of cryopreserved/thawed human ovarian tissue obtained from female cancer patients (*n* = 11) were xenotransplanted into a subcutaneous neck pouch of 6-week-old ovarectomized SCID mice (*n* = 33) for 4 (*n* = 18) and 12 (*n* = 15) weeks.

**Result:**

Thirty-two out of 33 mice survived the entire observation periods. Graft recovery rate was 95.58 % (65 of 68 grafts). The percentages of primordial follicles after 4 weeks (*P* < 0.001) and 12 weeks (*P* = 0.009) of grafting were significantly lower in comparison to pregraft controls. The percentage of secondary follicle was significantly higher after 4 weeks of grafting (*P* = 0.018) and after 12 weeks (*P* = 0.001) of grafting in comparison to pregraft controls. Ki67 immunohistochemistry showed that proliferative follicles were significantly higher after 4 and 12 weeks of grafting compared to pregraft controls (*P* < 0.001). All follicles analyzed by TUNEL staining appeared healthy after xenotransplantation. The expression level of PTEN was reduced by 2.47-fold after 4 weeks of xenotransplantation, and this result was significant when 2^−ΔCt^ were analyzed (*P* = 0.042).

**Conclusion:**

The higher proportion of growing follicles compared to resting follicles observed after xenotransplantation is most likely due to downregulation of PTEN gene expression followed by acceleration of follicular recruitment.

**Electronic supplementary material:**

The online version of this article (doi:10.1007/s10815-016-0769-2) contains supplementary material, which is available to authorized users.

## Introduction

The numbers of long-term cancer survivors are steadily increasing during the last decades leading to an actual 5-year survival rate for children of 79 % [1]. Several factors such as early cancer detection, advanced diagnostic tools, and aggressive cancer therapies have been contributing to this improvement. Therefore, due to the increasing life expectancy in cancer patients, the late side effects caused by high dose chemotherapies and radiotherapies are currently gaining more attention. One of the common and serious long-term side effects related to cancer therapy in female patients is premature ovarian failure (POF). Chemotherapeutic agents and irradiation are known to induce POF through destruction of ovarian follicular reserve by different mechanisms depending on the age of the patients, as well as on the type and the dose of cytotoxic agents [2–6].

To overcome this problem, several multidisciplinary strategies have been established to preserve female fertility [7–11]. One of the important developments in fertility preservation is ovarian tissue cryopreservation and autotransplantation. This method might be the only available method for prepubertal girls. Furthermore, it can be offered to patients immediately without delay in cancer therapy [9]. So far, there are more than 60 live births reported worldwide after autotransplantation of cryopreserved/thawed human ovarian tissue [12–22]. Data from three fertility preservation centers showed that endocrine ovarian function can be restored in 93 % of patients after autotransplantation [23]. Pregnancy rates after 111 cases of ovarian tissue autotransplantation in 5 fertility preservation centers were 29 % [24]. However, clinical data showed decreased ovarian reserve and low response to ovarian stimulation in patients after ovarian tissue autotransplantation [25, 26]. This decreased ovarian reserve after transplantation was proven by the low concentrations of anti-Mullerian hormone (AMH) and inhibin B as well as high levels of follicle-stimulating hormone (FSH) [25–27]. This indicates that further studies are needed to improve the outcome of ovarian tissue cryopreservation followed by autotransplantation.

One accepted experimental approach to evaluate functionality of cryopreserved human ovarian tissue is xenotransplantation into severe combined immunodeficient (SCID) mice [28]. Several studies showed a considerable decline in follicular density and an increase of follicular growth after xenotransplantation [29–32]. However, the mechanisms behind this rapid follicular recruitment and subsequent follicular loss after xenotransplantation are still unclear. Especially molecules regulating cell growth and organ size are currently being investigated as published by Kawamura et al. [14]. Furthermore, Roness et al. [33] hypothesized that the increase of follicular growth might be caused by disruption of follicular growth regulation leading to “ovarian follicle burnout.” The same authors observed this phenomenon in a mouse model, after administration of the gonadotoxic agent cyclophosphamide as well as after xenotransplantation of untreated bovine ovarian tissue [34, 35]. In addition to that, Silber et al. [20] observed an “over-recruitment” of resting follicles after ovarian tissue transplantation indicated by the increase of AMH to above normal levels in the recipient compared to the donor.

One of the important pathways for maintenance of follicular dormancy is the phosphoinositide 3-kinase (PI3K)/phosphatase and tensin homolog deleted on chromosome 10 (PTEN)/protein kinase B (Akt) pathway [36–38]. PTEN has been shown to be an important inhibitor of follicular growth initiation [39]. Oocyte-specific deletion of PTEN in mice led to over-activation of primordial follicles and subsequently to POF [39, 40]. In addition, it has been shown that PTEN inhibitors can cause follicular activation of murine follicles in vivo and of human follicles in vitro [41–43].

The aim of this study was to evaluate the mechanisms involved in follicular recruitment after xenotransplantation of human ovarian tissue in SCID mice and to investigate the impact of transplantation on follicular growth and depletion.

## Materials and methods

### Ethical approval

The use of human ovarian tissue samples in this study was approved by the Ethical Committee of the Medical University of Innsbruck. The animal experimentation in this study was performed under the approval from the Federal Ministry of Science and Research of Austria.

### Human ovarian tissue

Human ovarian tissue samples used in this study were obtained from 11 cancer patients with informed consent. The age of the patients at the time of ovarian tissue cryopreservation ranged from 20 to 39 years (mean age 27.0 years). AMH was determined with a commercially available ELISA kit (AMH Gen II ELISA, Beckman Coulter, Germany) before laparoscopic retrieval of ovarian tissue in all patients except one. Patient characteristics are presented in Table 1.

**Table 1:** Patients’ characteristics

No.	Diagnosis	Age at cryopreservation	AMH prior to surgery (μg/l)	Chemotherapy prior to surgery (Y/N)
1	Morbus Hodgkin	26	1.06	N
2	Breast Cancer	33	2.66	N
3	Morbus Hodgkin	22	Not applicable^a^	N
4	Breast cancer	33	0.86	N
5	Breast cancer	27	2.14	N
6	Morbus Hodgkin	27	1.62	Y
7	Breast Cancer	39	<0.17	N
8	ALL	20	2.66	Y
9	B-NHL	22	0.33	Y
10	AML	23	<0.17	Y
11	NHL	25	2.70	N

In 10 out of 11 patients, AMH levels were measured before the laparoscopical retrieval of ovarian tissue for cryopreservation. Seven patients did not receive chemotherapy before ovarian tissue cryopreservation and four patients received chemotherapy before ovarian tissue cryopreservation. In one patient^a^, AMH level (<0.17 μg/l) was determined after the surgery and after the first cycle of chemotherapy

### Animals

Female CB17/lcrHanHsd SCID mice were purchased from Harlan Laboratories, Italy, and delivered at 5 weeks of age. They were housed in a temperature- and light-controlled environment (22 °C and 12-h light/12-h dark) in individually ventilated cages (IVC) with high-efficiency particulate air filters, at the animal care facility of the Medical University of Innsbruck. Animals were supplied with water and food ad libidum, and a maximum of six animals were housed per cage. Animals were allowed to acclimatize for 1 week.

All of the procedures related to the animals were performed under a laminar flow-hood. Thirty-three 6-week-old mice were used in this study. Five independent repeats of the complete experiment were carried out.

### Collection of human ovarian tissue

Human ovarian tissue samples were laparoscopically retrieved and transferred to the laboratory of the Department of Gynecological Endocrinology and Reproductive Medicine, Medical University of Innsbruck, in Leibovitz medium (L-15, Invitrogen, Germany) within a 50-ml polystyrene conical tube (BD Falcon, BD Biosciences, Austria).

### Human ovarian tissue cryopreservation

Cortical strips from human ovarian tissue samples (~10 × 5 × 2 mm) were prepared under sterile conditions in Leibovitz medium. The cryopreservation procedure was performed using a slow-freezing protocol. Ovarian tissue samples were transferred to 2.0 ml cryovials (Nunc, Germany) containing 1.7 ml of cryopreservation medium (OvarStore, Gynemed, Germany) and placed into an automated, computer-controlled freezing system (IceCube 14S, Sy-Lab, Austria). The initial cooling rate was −2 °C/min to −7 °C. At this temperature, manual seeding was performed. After keeping the tissue for 10 min at this temperature, cooling was continued at the rate of −0.3 °C /min until −65 °C for approximately 220 min. Then cryovials were plunged into liquid nitrogen for storage.

### Human ovarian tissue thawing and preparation

Under aseptic conditions, cryovials were placed at room temperature for 30 s, and then transferred to a 37 °C water bath for 130 s. Afterwards, the tissue samples were washed stepwise in medium containing DPBS (Sigma–Aldrich, Austria), gentamicin (Sigma–Aldrich, Austria), serum substitute supplements (Irvine Scientific, Austria), and decreasing sucrose concentrations (MP Biomedicals, Austria). The last washing step was performed in G-MOPS plus medium (Vitrolife, Austria). Thawed cortical strips were then dissected into 3-mm biopsies using standardized biopsy punches and immediately transported to the animal facility of the Medical University of Innsbruck within 50-ml falcon tubes containing 5 ml pre-warmed G-MOPS medium at 37 °C.

### Xenotransplantation procedure

Mice were anesthetized with 0.1 mg/g body weight ketamine hydrochloride (Ketasol, Gräub, Ogris Pharma, Austria) and 0.015 mg/g body weight of xylazine (Xylasol, Ani Medica Ogris Pharma, Austria) intraperitoneally [44]. The average body weight was 16.66 g and ranged from 14.50 to 18.40 g. The surgery was performed on a 37 °C warming plate for animal surgery (Vettech, UK). The eyes of the mice were protected with an eye protection gel (Siccaforte, Agepha Pharmaceutical, Austria). Bilateral ovarectomy was performed via a dorsomedian incision. Excised mouse ovaries were examined under a stereomicroscope (MZ6, Leica, Austria) to ensure complete ovarectomy. After ovarectomy, two 3-mm thawed human ovarian tissue pieces were xenotransplanted into a subcutaneous neck-pouch and the grafts were fixed with two 6/0 nylon sutures (Prolene, Ethicon, Johnson & Johnson Medical Company, Austria). The peritoneum and skin incisions were closed with a 6/0 nylon suture. Post-operative analgesia was assured by adding 25 μg tramadol (Tramabene, Ratiopharm, Germany) per milliliter of drinking water. After grafting, the SCID mice were randomized to be observed for either for 4 (*n* = 18) or 12 (*n* = 15) weeks. At the end of each observation period, the animals were sacrificed. The grafts were recovered and immediately fixed in 3.6 % formalin. Ovarian tissue samples directly after thawing served as pregraft controls.

All procedures were carried out under a laminar flow hood in order to maintain sterile conditions.

### Follicle counting

Grafts were fixed in formalin and embedded in paraffin for histological assessment. The paraffin blocks were cut into serial sections of 3–5 μm. Every 12th section was stained with Hematoxylin and Eosin (H&E) for follicle classification and counting [45]. The sections in between were used for immunohistochemistry and RNA isolation. Follicular development was assessed by evaluating the follicular stages using a light microscope (BX 40; Olympus, Germany) as described by Myers et al. [46]. Briefly, a primordial follicle was defined by an oocyte surrounded by one layer of flattened pregranulosa cells, a primary follicle by an oocyte surrounded by one layer of cuboidal granulosa cells, and a secondary follicle by two or more layers of granulosa cells. An antral follicle was defined by antrum formation. In the case of intermediate stages between primordial and primary follicles, those follicles showing at least one cuboidal granulosa cell were classified as primary follicles. Only follicles with a visible nucleus in the oocyte were counted [47].

### Follicular proliferation

Follicular proliferation was immunohistochemically evaluated by the detection of the expression of the Ki67-protein in granulosa cells. Ki67 is expressed during proliferative cell phases (G1, S, G2, and mitosis), but not during the resting cell phase (G0).

Ki67 immunostaining was performed within an automated slide staining system (Benchmark Ultra, Ventana, USA) using a primary monoclonal rabbit antibody raised against human Ki67 (CONFIRM anti-Ki67, Ventana, Roche GmbH, USA), a secondary goat anti- rabbit antibody (HRP system, Ventana, Roche GmbH, USA), and the 3,3-diaminobenzidine tetrahydrochloride (DAB) chromogen (ultraView DAB detection Kit, Ventana, Roche GmbH, USA). Nuclei were counterstained with hematoxylin. Human breast carcinoma tissue was used as positive control. Only sections where follicles were detected in the adjacent H&E-stained sections were analyzed.

Follicles with at least one granulosa cell stained positive with Ki67 were considered to be proliferative follicles [48].

### Follicular apoptosis

To analyze apoptosis after xenotransplantation, terminal deoxynucleotidyl transferase-mediated dUTP nick end labeling (TUNEL) assay (DeadEndTM Colorimetric TUNEL System, Promega, Austria) was performed according to the manufacturer’s instructions, with 3,3-diaminobenzidine tetrahydrochloride (DAB) as chromogen. Mayer’s hemalaun solution (Merck Millipore, Germany) was used for counterstaining. Only sections where follicles were detected in the adjacent H&E-stained sections were analyzed. The TUNEL staining results were evaluated by three independent observers.

Follicles consisting of ≥49 % TUNEL positive granulosa cells as well as follicles containing a TUNEL positive oocyte were classified as atretic follicles [49]. Sections without transferase enzyme and human lymph node sections treated with DNAse served as negative and positive control, respectively.

### PI3K pathway analysis

#### PTEN gene expression

Total ribonucleic acid (RNA) was isolated from 19 grafts bearing follicles obtained from 8 patients. Twenty sections per paraffin block were used for RNA isolation with RNEasy FFPE kit according to manufacturer’s protocol (Qiagen, Germany). DNA digestion was performed using RNAse free DNAse (Promega, Austria).

RNA concentration was measured either with UV-spectrophotometer (GeneQuant 1300 Spectrophotometer, GE Healthcare, Austria) or with a fluorescence staining kit (Quant-iT Ribogreen RNA reagent, Life Technologies, Germany). RNA was stored at −80 °C. Reverse transcription was performed with 50 ng of isolated RNA using the Omniscript reverse transcriptase (Qiagen, Germany) and random hexanucleotide primer (Carl Roth, Austria). Quantitative PCR (qPCR) was performed using the Maxima SyBr Green/ROX qPCR master mix (Thermo Scientific, Austria). The following primers were used: PTEN (forward: PTEN, 5′-TGACAATCATGTTGCAGCAATTC-3′, reverse: 5′-CACCAGTTCGTCCCTTTCCA-3′) as the target gene [50]. Actin (forward: 5′-ACT GGG ACG ACA TGG AGA AG-3′, reverse: 5′-GGG GTG TTG AAG GTC TCA AA-3′) was used as housekeeping gene.

qPCR was performed in 96-well plates (Peqlab, Austria) using a BioRad iCycler (BioRad, Austria). The PCR profile was started with an initial incubation at 95 °C for 10 s, followed by 40 cycles with denaturation at 95 °C for 5 s, and annealing and extension at 60 °C for 45 s. All qPCR measurements were performed in triplicates and samples without reverse transcriptase served as a negative control.

qPCR data were analyzed with iQ5 software (BioRad, Austria), and the efficiency of each qPCR run was evaluated with LinRegPCR data analysis program (version 2013.1; http://LinRegPCR.nl) [51, 52].

PTEN gene expression level was calculated by subtracting the actin CT values from PTEN CT values (**Δ**Ct). Relative quantitative analysis of PTEN gene expression was carried out using the **ΔΔ**Ct method [53]. Fold change difference of PTEN expression was determined as 2^−(**Δ**Ct 4 weeks–**Δ**Ct pregraft)^.

#### PTEN immunohistochemistry

To analyze the protein expression of PTEN, immunohistochemistry was performed. Formalin-fixed and paraffin-embedded tissue were cut into 3–5-μm-thin sections and heated for 20 min at 70 °C. PTEN immunostaining was performed within an automated slide staining system (Benchmark Ultra, Ventana, USA) with a primary monoclonal rabbit anti-PTEN antibody (Clone D4.3, 1:250, Cell Signaling, USA), a secondary goat anti- rabbit antibody (HRP system, Ventana, Roche GmbH, USA), and the DAB chromogen (ultraView DAB detection Kit, Ventana, Roche GmbH, USA). Nuclei were counterstained with hematoxylin. Human colon tissue was used as positive control and sections stained without any primary antibody was used as negative control.

### Statistics

Statistical analysis was performed using the SPSS software package (version 20; SPSS, Inc., USA). All data are represented as mean ± SEM. The nonparametric Mann–Whitney *U* test was performed to compare follicle counts. A paired-sample *t* test was performed to compare PTEN gene expression. Immunohistochemical results from Ki67 were analyzed with chi-square test. Statistical significance was confirmed by *P* values <0.05.

## Results

### Macroscopic evaluation of the grafts

Thirty-two of 33 mice (96.97 %) survived throughout the whole observation period. Sixty-five out of 68 grafts (95.58 %) were successfully recovered from all groups. Two grafts showed a macroscopically visible antral cavity after 12 weeks of grafting.

### Rapid follicular recruitment after xenotransplantation of cryopreserved/thawed human ovarian tissue

#### Follicle counting

**Fig. 1:**
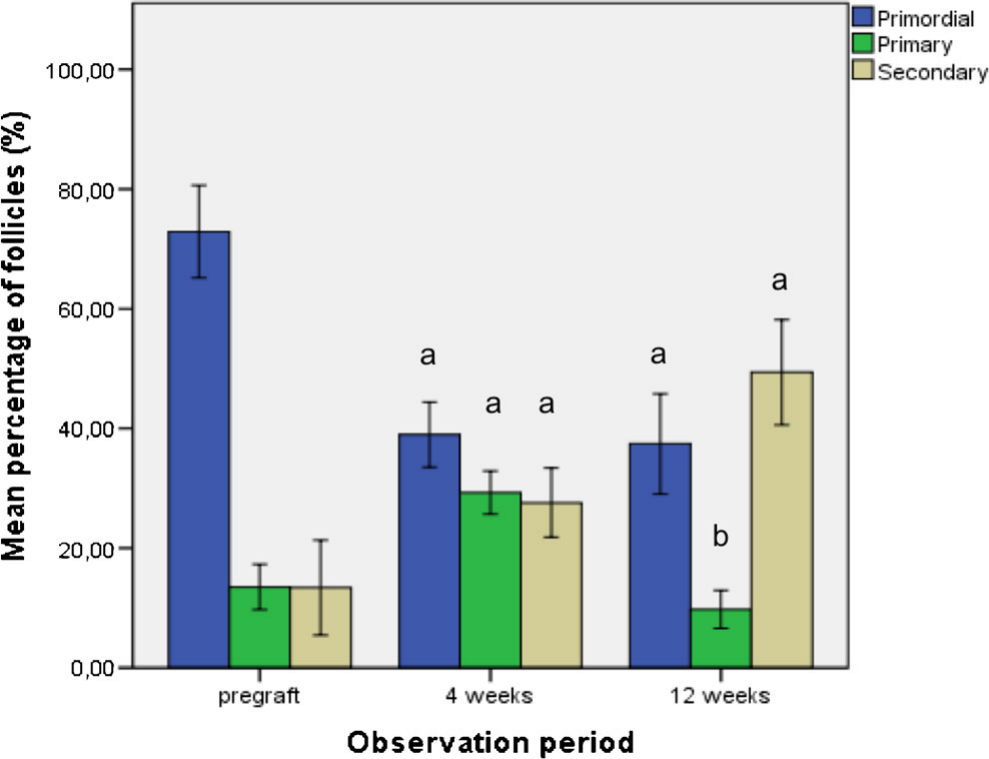
Follicles were classified and counted in pregraft control, 4-week grafts, and 12-week grafts. Total follicles per biopsy as well as percentages of primordial, primary, and secondary follicles are presented in mean ± SEM. Antral follicles are not represented in the figure and the table because they formed only 0.49 % (5/1014) of all follicles observed. *a* Significant differences between the two observation periods and pregraft control (*P* < 0.05). *b* Significant differences between 4 and 12 weeks of grafting (*P* < 0.05)

A total of 1014 follicles were counted within 58 biopsies. Twenty-six biopsies did not contain any follicles and were excluded from further calculations. Pregraft controls (*n* = 17) contained 669 follicles, 4-week grafts (*n* = 24) contained 183 follicles, and 12-week grafts (*n* = 17) contained 162 follicles. The percentage of primordial follicles after 4 weeks of grafting (38.95 ± 3.94 %) was significantly lower in comparison to pregraft controls (72.88 ± 5.93 %, *P* < 0.001). The percentage of primordial follicles after 12 weeks of grafting (37.42 ± 5.83 %) was also significantly lower (*P* = 0.009) compared to pregraft controls. The percentage of primary follicles was also significantly different after 4 weeks (29.29 ± 2.60 %, *P* = 0.009) compared to pregraft controls (13.48 ± 2.92 %). However, the percentage of primary follicles was not significantly different after 12 weeks of grafting (9.74 % ± 2.22 %) compared to pregraft control (*P* = 0.259). The percentage of secondary follicles was significantly increased after 4 weeks (27.60 ± 4.16 %, *P* = 0.018) and 12 weeks (49.40 ± 6.14 %, *P* = 0.001) of grafting compared to pregraft controls (13.37 ± 6.09 %). There were two antral follicles observed in the pregraft control, one after 4 weeks of grafting, and two after 12 weeks of grafting. There was a significant difference in the percentage of primary follicles (*P* = 0.001) between the two observation periods after grafting (Fig. 1).

### Follicular growth

A total of 196 follicles from 10 patients were analyzed for Ki67 immunohistochemistry. The percentage of all Ki67-positive follicles after 4 (70.59 %, *P* < 0.001) and 12 (81.82 %, *P* < 0.001) weeks of grafting was significantly higher compared to pregraft controls (11.02 %). The percentage of Ki67-positive primordial follicles increased from 10.28 % in pregraft controls to 70.83 % after 4 weeks of grafting (*P* < 0.001) and to 70.59 % after 12 weeks of grafting (*P* < 0.001). The percentage of Ki67 positive primary follicles was not significantly higher after 4 weeks of grafting (50 %, *P* = 0.205) compared to pregraft controls (18.18 %), but it was significantly higher after 12 weeks of grafting (72.73 %, *P* = 0.015) compared to pregraft controls (18.18 %). All detected secondary follicles were Ki67-positive regardless of the observation period. Two antral follicles were found after 12 weeks of grafting and both were Ki67 positive. There was no significant difference in the percentage of Ki67 positive follicles of all stages between 4 and 12 weeks of grafting (Table 2 and supplementary Fig. 2).

**Table 2:** Percentage of Ki67 positive follicles in pregraft controls, 4-week grafts, and 12-week grafts

Follicle stage	Pregraft	4 weeks	12 weeks
Primordial	10.28 %	70.83 %^a^	70.59 %^a^
	(11/107)	(17/24)	(12/17)
Primary	18.18 %	50 %	72.73 %^a^
	(2/11)	(3/6)	(8/11)
Secondary	–	100 %	100 %
	–	(4/4)	(14/14)
Antral	–	–	100 %
	–	–	(2/2)
Total Ki67 positive follicles	11.02 %	70.59 %^a^	81.82 %^a^
	(13/118)	(24/34)	(36/44)

Percentage of primordial, primary, secondary, and antral follicles showing Ki67 immunostaining in pregraft controls and after 4 and 12 weeks of grafting, respectively. The total number of Ki67-positive follicles as well as the total number of all analyzed follicles are given in brackets

^a^Significant difference between the two observation periods compared to the pregraft control (*P* < 0.05)

### Follicular apoptosis

In order to detect signs of apoptosis, 96 follicles from 9 patients were analyzed. In pregraft controls and after 4 weeks of grafting, none of the follicles were TUNEL positive. After 12 weeks of grafting, one secondary follicle showed one TUNEL-positive granulosa cell (supplementary Fig. 3).

### Downregulation of PTEN expression after xenotransplantation of cryopreserved/thawed human ovarian tissue

#### PTEN gene expression

**Fig. 2:**
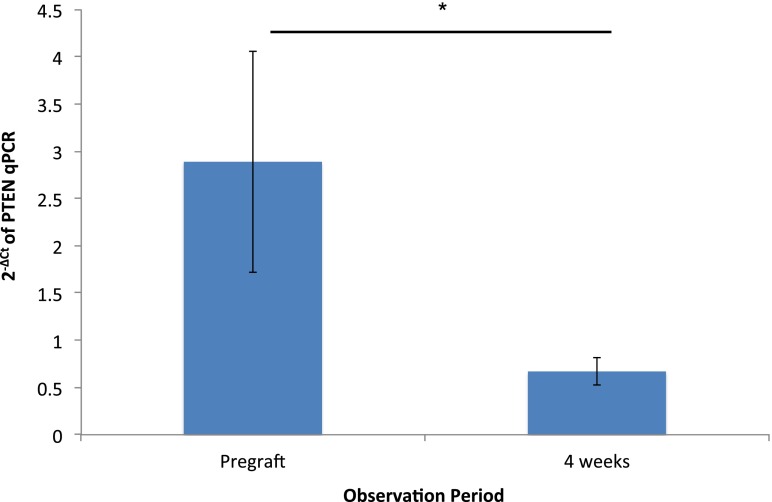
Relative expression of the PTEN gene after xenotransplantation compared to pregraft control. Figure shows 2.47-fold reduction of the PTEN gene expression after 4 weeks of grafting. This difference reached significance when 2^−ΔCt^ were analyzed. Data are presented in mean ± SEM

PTEN expression was analyzed in 8 pregraft controls and 11 4-week-grafts obtained from 8 patients. Genomic DNA contamination was negative in all RNA samples. The relative gene expression of PTEN was compared between pregraft controls and 4-week grafts after normalization using actin as the housekeeping gene. The amplification efficiency of qPCR was higher than 1.8 in all measurements. The expression of PTEN was reduced by 2.47-fold after 4 weeks of xenotransplantation (Fig. 2). This result was significant when 2^−ΔCt^ were analyzed (*P* = 0.042). There was no significant correlation between the reduced numbers of follicles and PTEN expression as shown in supplementary data (supplementary Fig. 5).

#### PTEN immunohistochemistry

PTEN protein expression was detectable in theca cells and granulosa cells from growing follicles, as well as in the nuclei and cytoplasm of all oocytes (supplementary Fig. 6). In granulosa cells of primordial follicles, PTEN protein was either absent or weakly expressed.

## Discussion

This study examines for the first time the molecular mechanisms that may be involved in the increased follicular recruitment after xenotransplantation of cryopreserved/thawed human ovarian tissue. The data, compiled from a considerable number of patients, show that the observed acceleration of follicular growth is most likely due to downregulation of PTEN.

After 4 and 12 weeks of grafting, histological evaluation revealed a pronounced decrease of primordial follicles accompanied by an increase of growing follicles. Only few granulosa cells and none of the oocytes were apoptotic after grafting. In addition, increased follicular recruitment was indicated by the increase of Ki-67-positive primordial and primary follicles after 4 and 12 weeks of xenotransplantation.

A corresponding downregulation of PTEN was detected with qPCR after grafting, without any significant correlation between the reduced numbers of follicles and PTEN expression as shown in supplementary data (supplementary Fig. 5).

PTEN is an important component of the PI3K pathway, which has been described to play a crucial role in follicular recruitment [36]. The deletion of PTEN in mice has been shown to result in premature ovarian failure due to the complete depletion of primordial follicles [39]. In vitro culture studies in human ovarian tissue by Novella-Maestre et al. [54] showed that PTEN inhibition enhances the activation of primordial follicles and increases the population of growing follicles without inducing apoptosis in follicles or in the surrounding stroma. Other in vitro studies by McLaughlin et al. [42] demonstrated that the addition of the PTEN inhibitor bpV(HOpic) alters human ovarian follicular growth by inducing the initiation of primordial follicle recruitment to the secondary stage. Additionally, Li et al. [43] proved that the administration of a PTEN inhibitor bpV(HOpic) and a PI3K-activating peptide 740Y-P to ovarian tissue could promote follicular development and oocyte maturation.

This ovarian follicle “burnout” caused by dysregulation of the PI3K pathway might be a universal phenomenon [33]. Kalich-Philosoph et al. demonstrated the accelerated growth of primordial follicles within 24 h after cyclophosphamide administration in rodents [34]. In accordance with our study, this was not due to apoptosis, but to activation of the PI3K pathway [34].

Additionally, Silber et al. [20] also observed a massive recruitment of resting follicles after transplantation of cryopreserved/thawed ovarian tissue shown by increased AMH levels.

Moreover, Silber et al. [55] hypothesized that resting follicles could undergo recruitment after an ovarian tissue fragmentation due to the release from the dense ovarian cortex, which physiologically might arrest follicle recruitment [55–57]. In addition, other studies showed that fragmentation of human ovarian tissue and incubation with Akt stimulators leads to the activation of dormant follicles in patients suffering from premature ovarian insufficiency [14, 19].

In conclusion, to our knowledge, our study confirms these results for the first time in vivo using human ovarian tissue from cancer patients in a xenotransplantation model. Certainly, further studies including shorter observation periods are needed in order to evaluate the starting point of follicular recruitment after xenotransplantation.

In addition, it is essential to investigate additional genes involved in the PI3K pathway, such as Akt, FOXO 3a, and mTOR in human ovarian tissue xenotransplantation. Furthermore, different graft sizes should be evaluated according to previous findings in bovine ovarian tissue.

Overall, the findings of this study might help improve post-transplantational outcomes, as loss of primordial follicles impairs longevity of the grafts after transplantation.

## Electronic supplementary material

Figure 2 (Supplementary). Immunohistochemical visualization of Ki67 in human ovarian tissue after xenotransplantation. Follicles without a visible nucleus of the oocyte were not taken into account. In the case of intermediate stages between primordial and primary follicles, those follicles showing at least one cuboidal granulosa cell were classified as primary follicles.

Figure 2 **a.** Ki67-positive primary follicle is indicated by an arrow (GIF 2725 kb)

High resolution image (TIF 10243 kb)

Figure 2 **b.** Ki67-positive antral follicle. (GIF 3156 kb)

High resolution image (TIF 7730 kb)

Figure 3 (Supplementary). TUNEL staining.

Figure 3 **a**. Positive Control for TUNEL staining: human lymph node tissue treated with DNAse was used as positive control for TUNEL staining; TUNEL positive cells were indicated by brown staining. (GIF 2907 kb)

High resolution image (TIF 7730 kb)

Figure 3 **b.** TUNEL positive granulosa cell (arrow) from a secondary follicle after human ovarian tissue xenotransplantation. (GIF 2989 kb)

High resolution image (TIF 10243 kb)

Figure 3 **c.** TUNEL negative follicles after xenotransplantation. (GIF 3373 kb)

High resolution image (TIF 3872 kb)

Figure 5 **(Supplementary). Correlation between numbers of follicles and PTEN gene expression.** After analyzing all of the samples’ results using Pearson’s correlation method, there was no significant correlation between numbers of follicles in the sections used for qPCR and PTEN gene expression after normalization with reference gene (P = 0.642). Figure shows the scattered plot of the correlation analysis. (PDF 21 kb)

Figure 6 **(Supplementary). Immunohistochemical visualization of PTEN in a human ovarian tissue graft.** Positive immunoreactivity of PTEN was indicated by brown staining of granulosa cells as well as staining of nuclei and cytoplasm of an oocyte. (GIF 2462 kb)

High resolution image (TIF 3872 kb)
