# Supplementary figures and images for: Follicular growth after xenotransplantation of cryopreserved/thawed human ovarian tissue in SCID mice: dynamics and molecular aspects
Source: J Assist Reprod Genet. 2016 Jul 27;33(12):1585–93. doi: 10.1007/s10815-016-0769-2 (PMC5171895; doi:10.1007/s10815-016-0769-2)

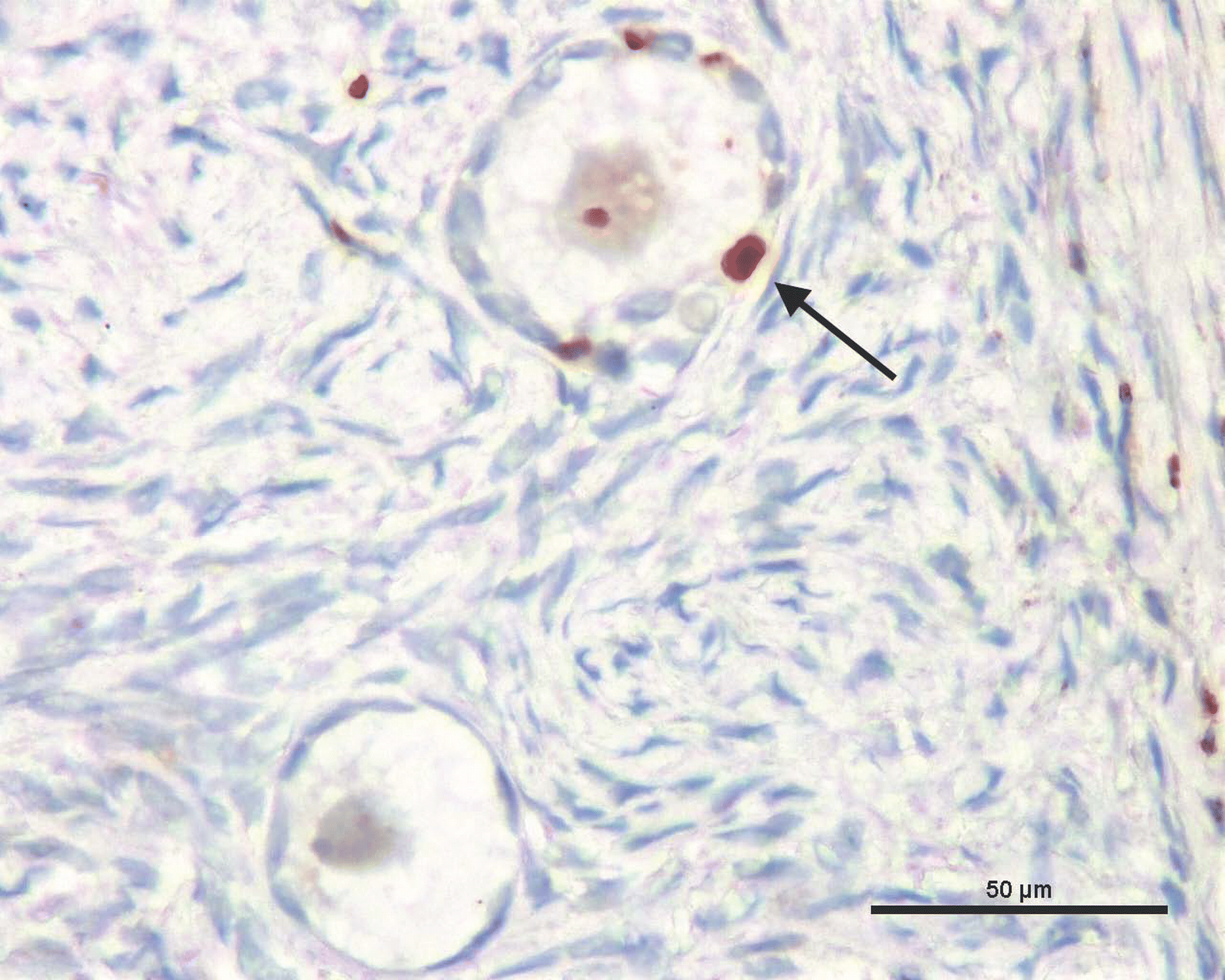

Supplement: Supplementary file 1 — a. Ki67-positive primary follicle is indicated by an arrow (GIF 2725 kb) [file 10815_2016_769_Fig3_ESM.gif]

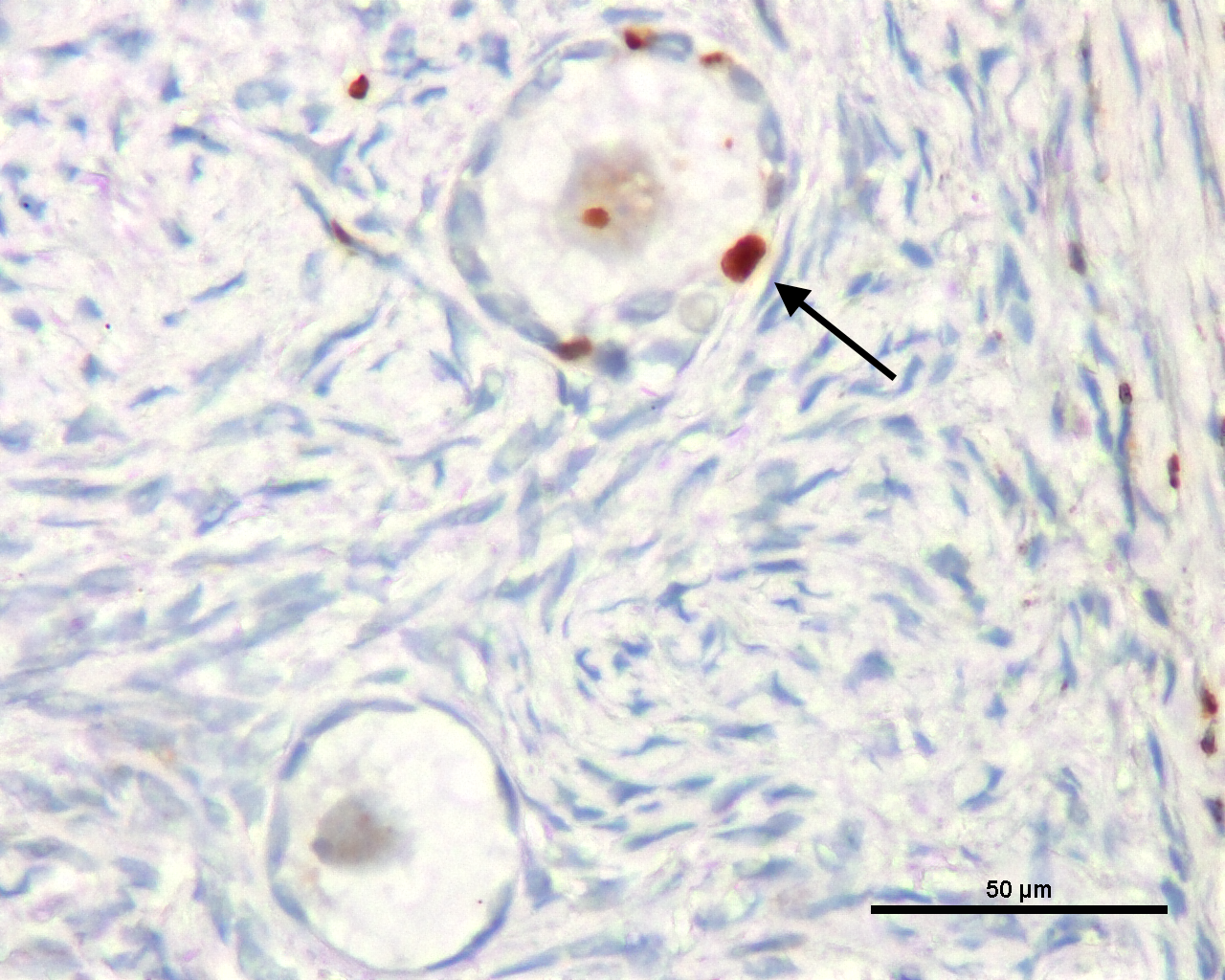

Supplement: Supplementary file 2 — High resolution image (TIF 10243 kb) [file 10815_2016_769_MOESM1_ESM.tif]

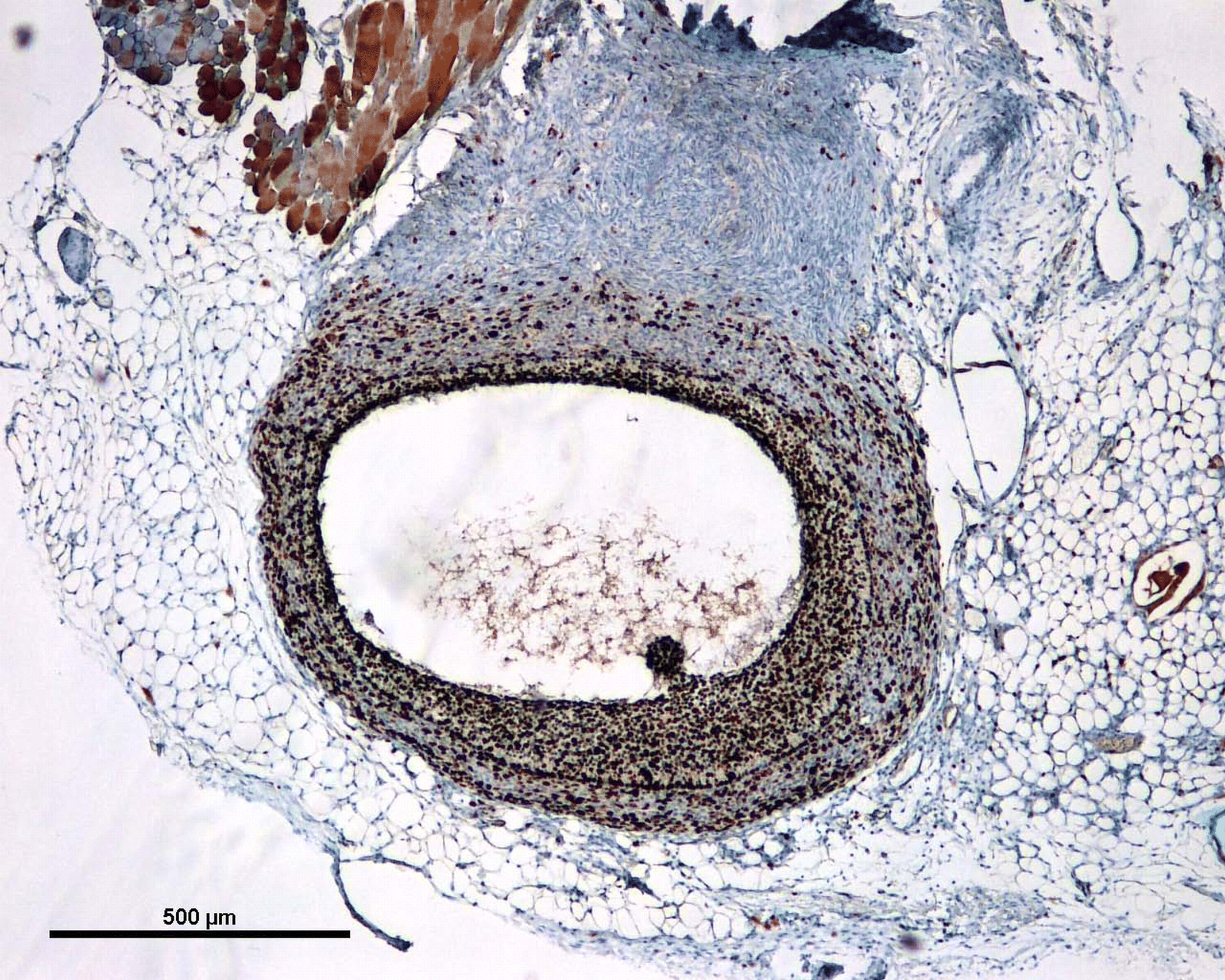

Supplement: Supplementary file 3 — b. Ki67-positive antral follicle. (GIF 3156 kb) [file 10815_2016_769_Fig4_ESM.gif]

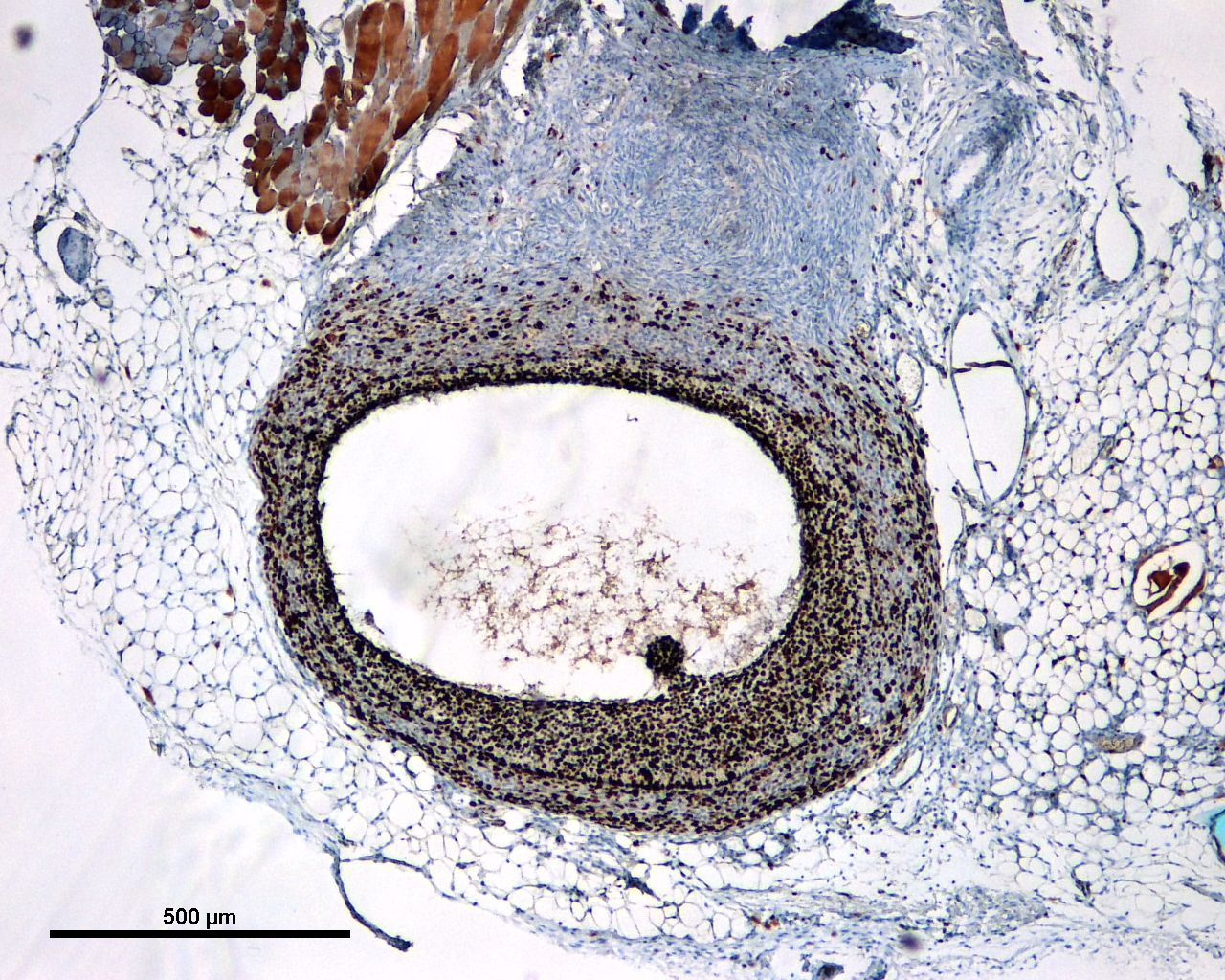

Supplement: Supplementary file 4 — High resolution image (TIF 7730 kb) [file 10815_2016_769_MOESM2_ESM.tif]

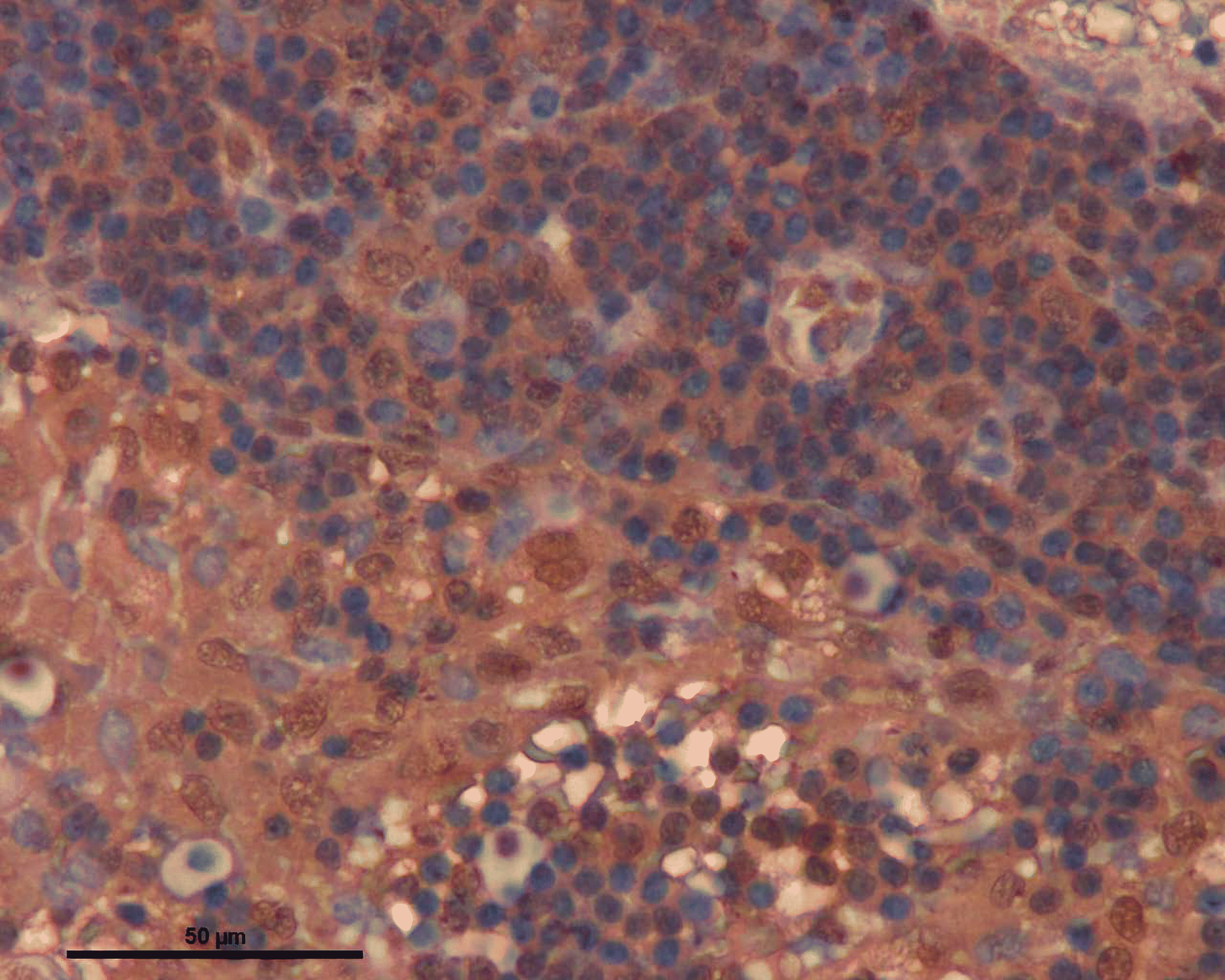

Supplement: Supplementary file 5 — a. Positive Control for TUNEL staining: human lymph node tissue treated with DNAse was used as positive control for TUNEL staining; TUNEL positive cells were indicated by brown staining. (GIF 2907 kb) [file 10815_2016_769_Fig5_ESM.gif]

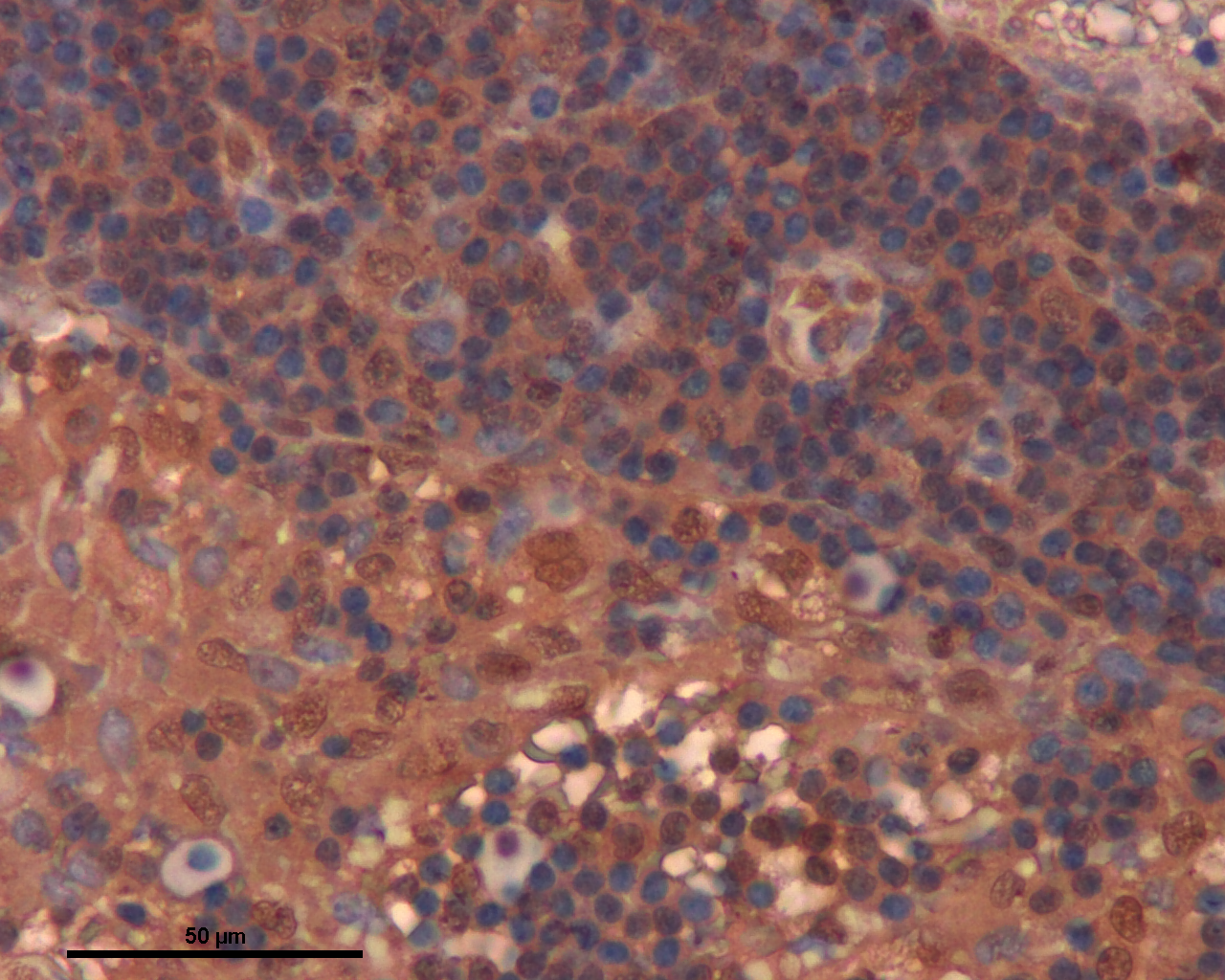

Supplement: Supplementary file 6 — High resolution image (TIF 7730 kb) [file 10815_2016_769_MOESM3_ESM.tif]

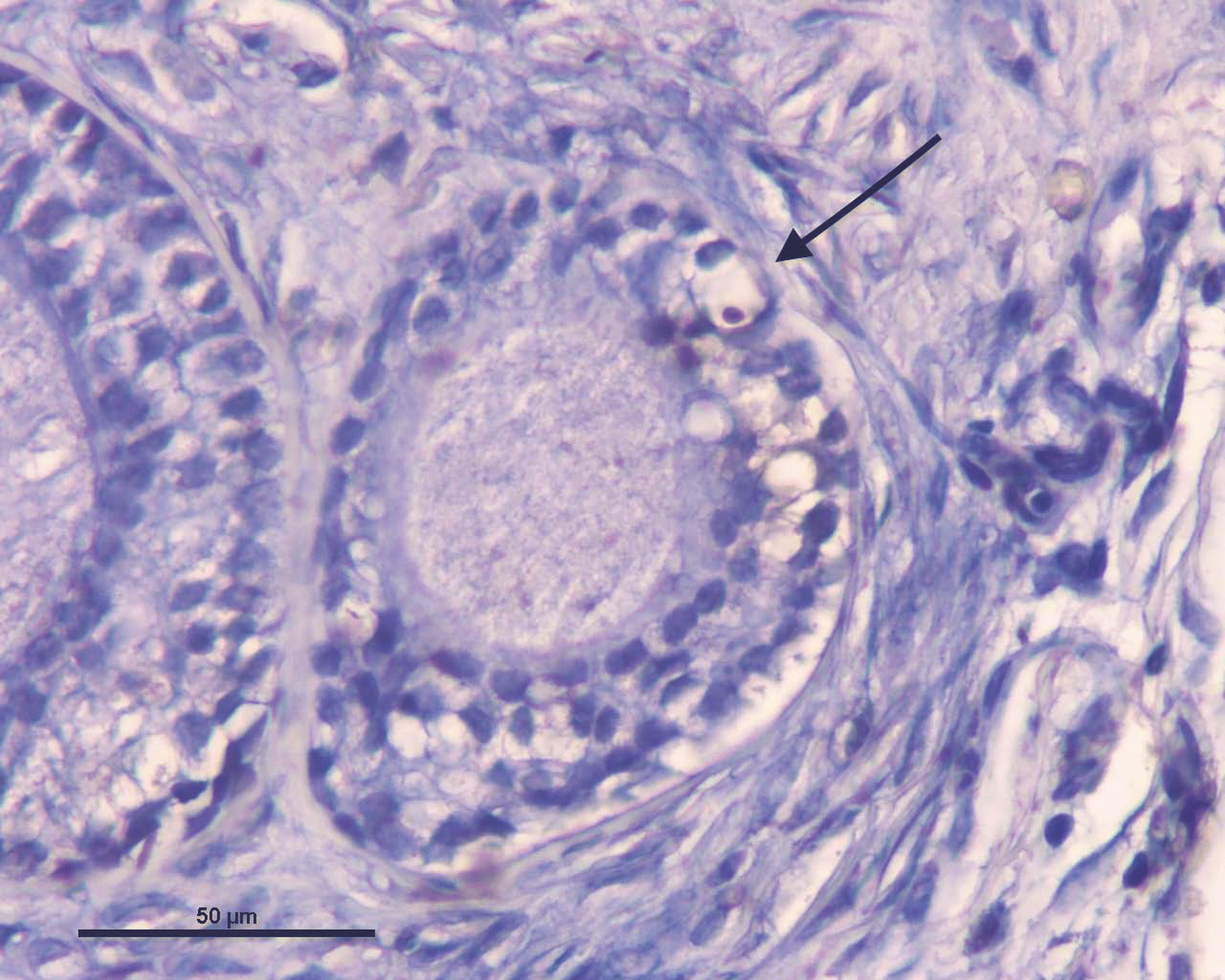

Supplement: Supplementary file 7 — b. TUNEL positive granulosa cell (arrow) from a secondary follicle after human ovarian tissue xenotransplantation. (GIF 2989 kb) [file 10815_2016_769_Fig6_ESM.gif]

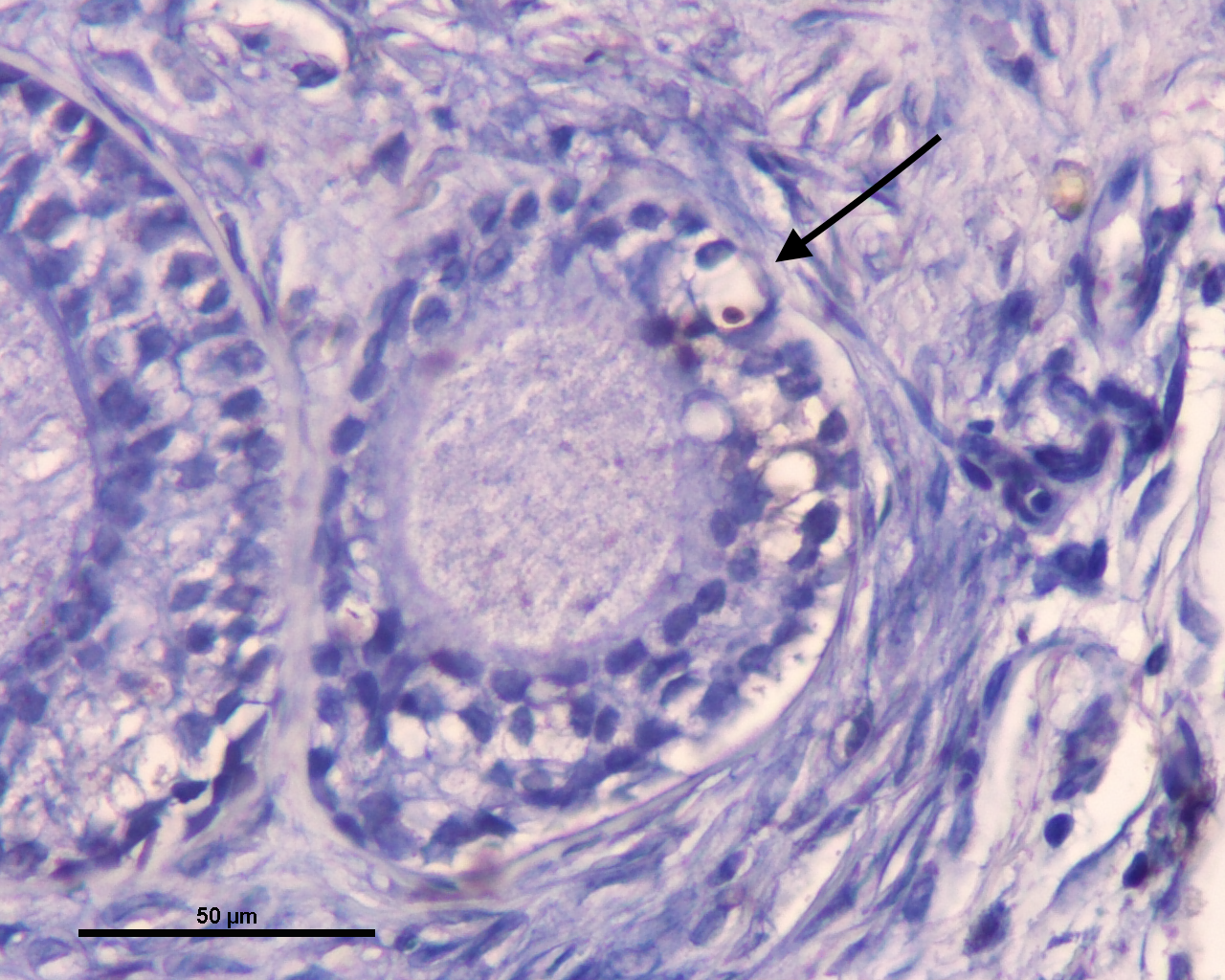

Supplement: Supplementary file 8 — High resolution image (TIF 10243 kb) [file 10815_2016_769_MOESM4_ESM.tif]

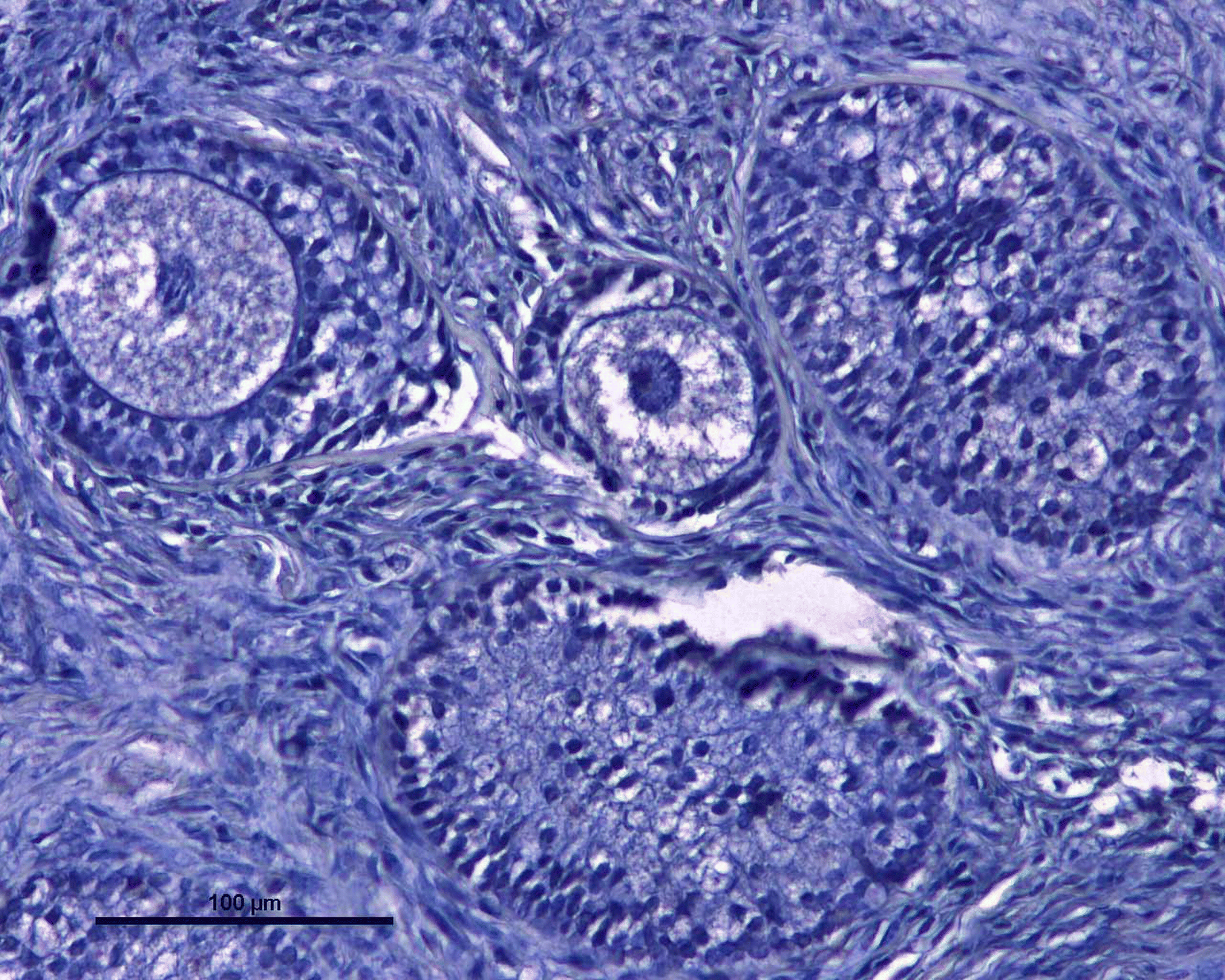

Supplement: Supplementary file 9 — c. TUNEL negative follicles after xenotransplantation. (GIF 3373 kb) [file 10815_2016_769_Fig7_ESM.gif]

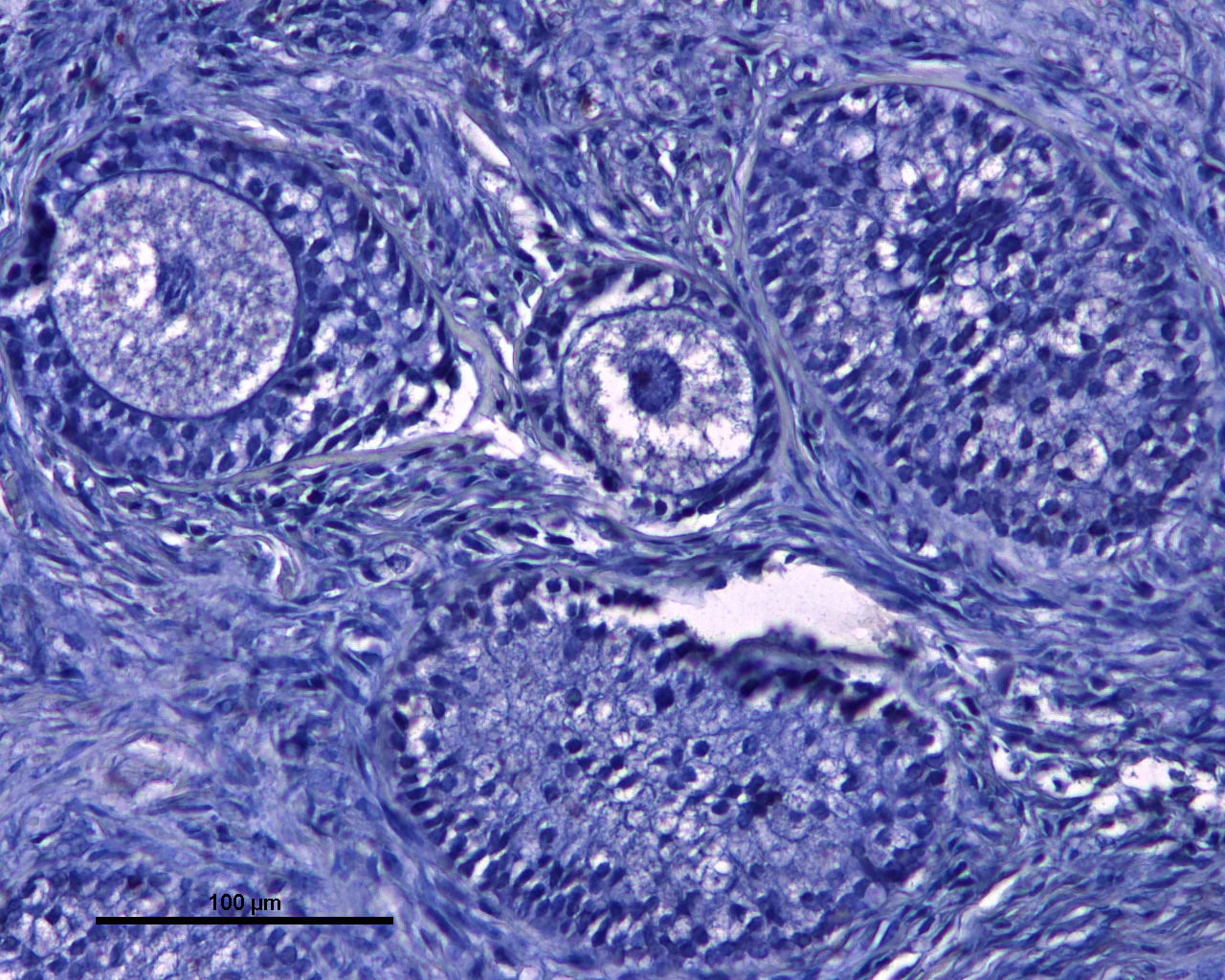

Supplement: Supplementary file 10 — High resolution image (TIF 3872 kb) [file 10815_2016_769_MOESM5_ESM.tif]

Delta CT

2.00  
1.00  
.00  
-1.00  
-2.00  
-3.00

.00

10.00

20.00

30.00

40.00

Total Follicles

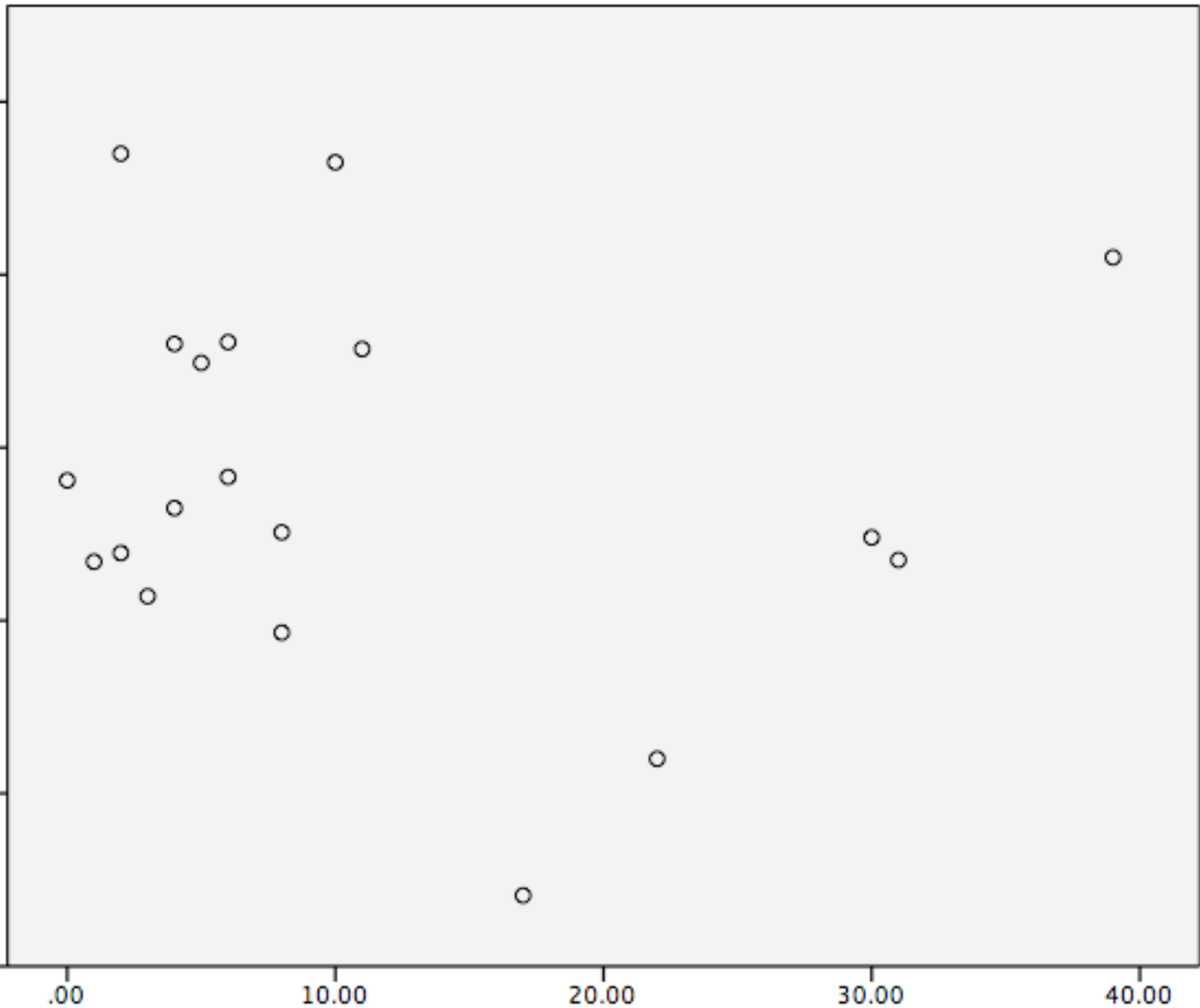

Supplement: Supplementary file 11 — (Supplementary). Correlation between numbers of follicles and PTEN gene expression. After analyzing all of the samples’ results using Pearson’s correlation method, there was no significant correlation between numbers of follicles in the sections used for qPCR and PTEN gene expression after normalization with reference gene (P = 0.642). Figure shows the scattered plot of the correlation analysis. (PDF 21 kb) [file 10815_2016_769_MOESM6_ESM.pdf]

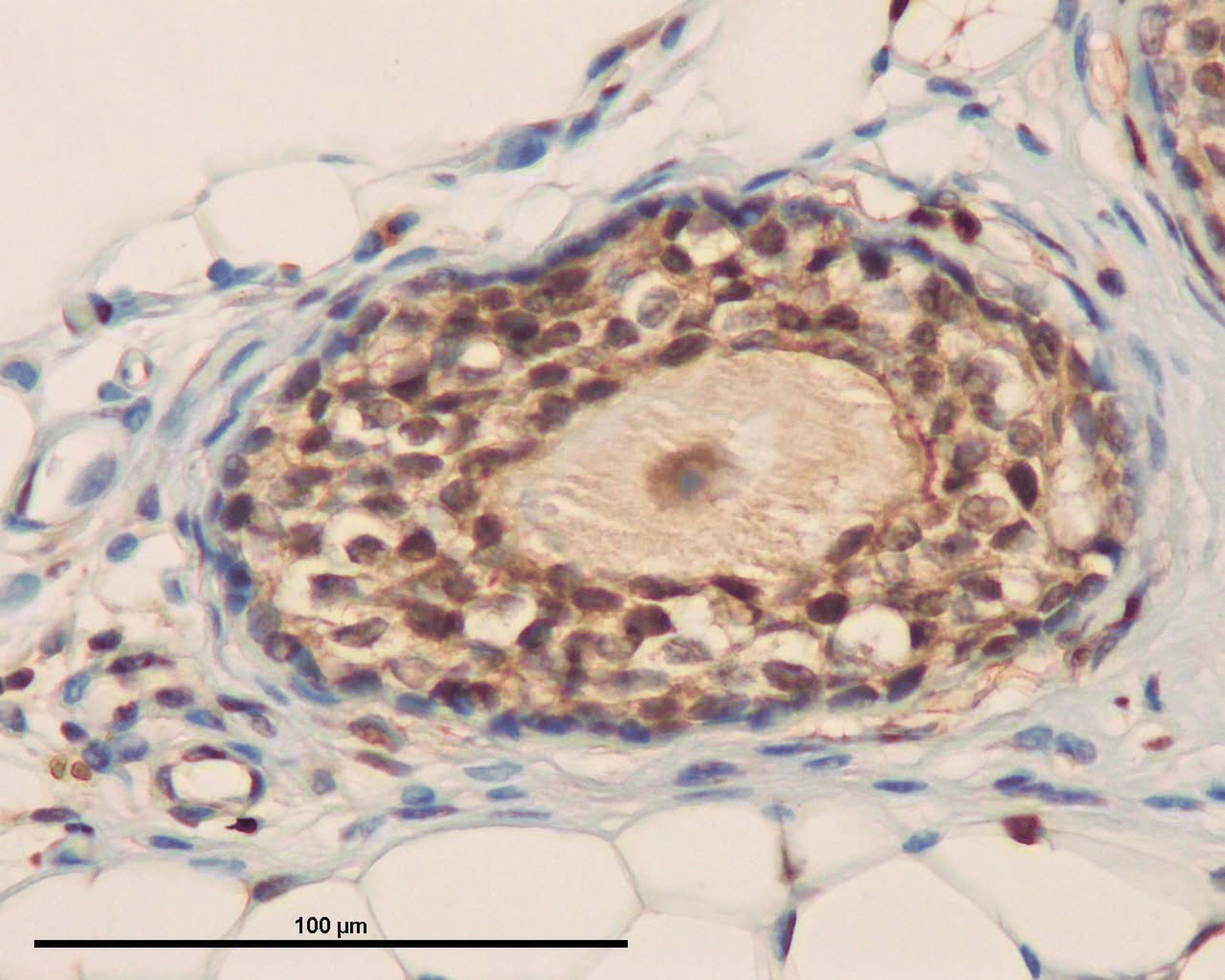

Supplement: Supplementary file 12 — (Supplementary). Immunohistochemical visualization of PTEN in a human ovarian tissue graft. Positive immunoreactivity of PTEN was indicated by brown staining of granulosa cells as well as staining of nuclei and cytoplasm of an oocyte. (GIF 2462 kb) [file 10815_2016_769_Fig8_ESM.gif]

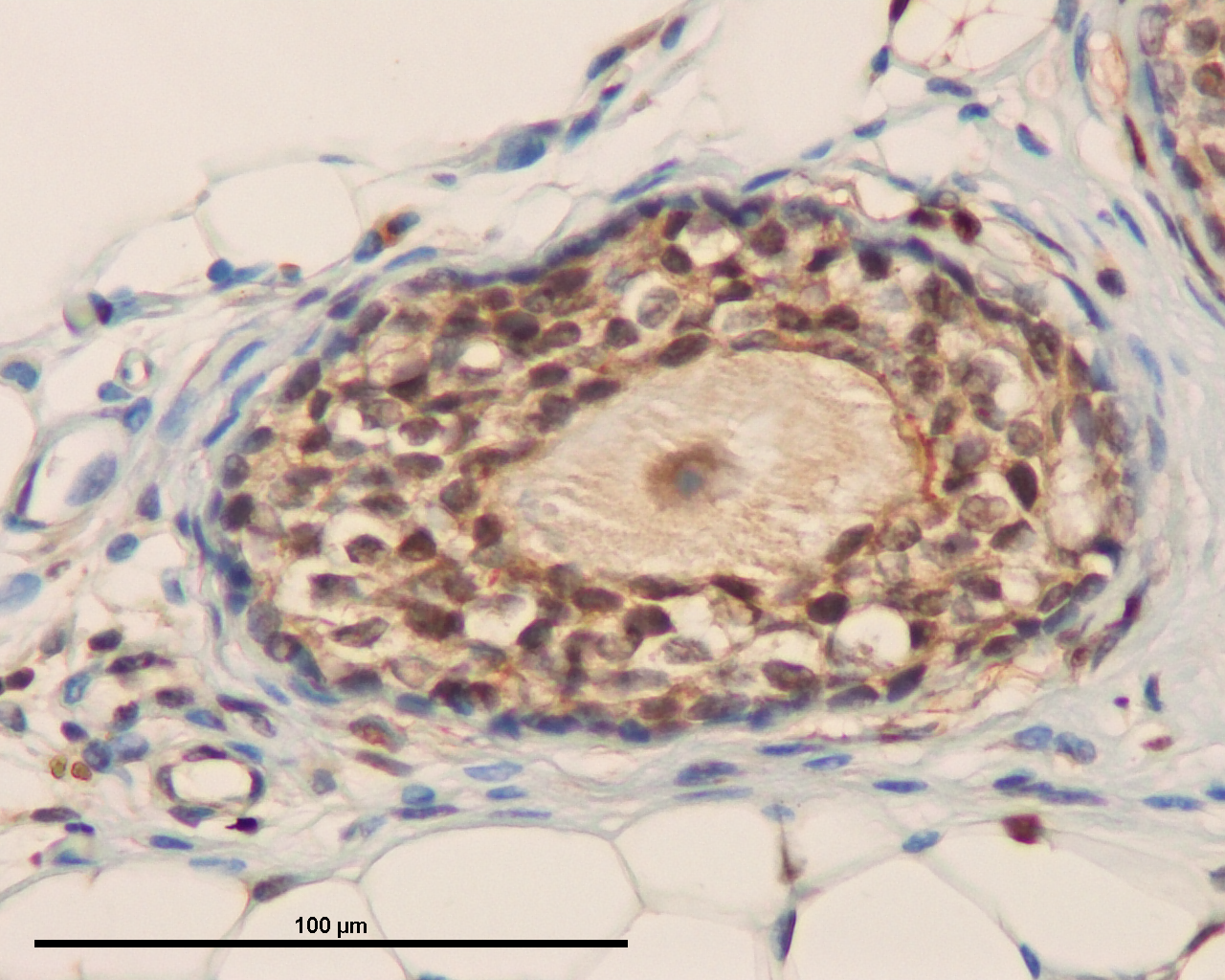

Supplement: Supplementary file 13 — High resolution image (TIF 3872 kb) [file 10815_2016_769_MOESM7_ESM.tif]
